# The association of molecular classification with fertility-sparing treatment of atypical endometrial hyperplasia and endometrial cancer: a retrospective study

**DOI:** 10.3389/fonc.2025.1506639

**Published:** 2025-04-15

**Authors:** Yuanyuan Wang, Tianjiao Lai, Danxia Chu, Jing Bai, Shuping Yan, Ruixia Guo

**Affiliations:** ^1^ Department of Gynecology, The First Affiliated Hospital of Zhengzhou University, Zhengzhou, Henan, China; ^2^ Department of Gynecology, The First Affiliated Hospital of Xinxiang Medical University, Weihui, Henan, China; ^3^ Department of Pathology, The First Affiliated Hospital of Zhengzhou University, Zhengzhou, Henan, China

**Keywords:** early-stage endometrial cancer, atypical endometrial hyperplasia, fertility-sparing treatment, molecular classification, complete response, partial response, progression disease

## Abstract

**Background:**

To evaluate whether molecular classification was associated with treatment response and recurrence in women with atypical endometrial hyperplasia (AEH) or early-stage endometrial cancer (EC) treated with progestin.

**Methods:**

A retrospective analysis of 116 patients (71 AEH and 45 EC) who received fertility-sparing therapy between 2010 and 2022 was performed. Tumors were classified via immunohistochemistry and gene sequencing into four subgroups: polymerase-ϵ (POLE)-mutated (POLEmut), tumor protein 53 (p53) wild type [p53wt; no specific molecular profile (NSMP)], mismatch repair deficient (MMRd), and p53 abnormal (p53abn). The primary outcome was complete response (CR) to hormone therapy. The secondary outcomes included the recurrence rate after CR and conception success. The treatment response to progestin and recurrence rate were compared across the four subgroups.

**Results:**

Of 116 patients, 10 (8.62%) were classified as POLEmut, 81 (69.82%) p53wt, 9 (7.76%) p53abn, and 16 (13.76%) MMRd. At the 12-month evaluation, 87 patients (75.00%) achieved CR (median treatment duration, 5.95 months; range, 1.6–12.9). Partial response (PR), stable disease (SD), and progressive disease (PD) rates were 6.89% (n = 8), 1.72% (n = 2), and 16.38% (n = 19), respectively. Patients with the p53abn and MMRd subtypes had lower CR rates (33.33% and 37.50%) and higher progression rates (44.40% and 37.50%) compared to other subgroups (*p* < 0.05). After 24-month follow-up, recurrence rates were markedly higher in the p53abn (100%) and MMRd (83.33%) subgroups versus the POLEmut (33.33%) and p53wt (17.39%) subgroups (*p* < 0.05). Among 56 (64.37%) patients attempting conception, the pregnancy rate of 38 patients who received *in vitro* fertilization-embryo transfer was 47.37% (18/38), and the pregnancy rate of 18 patients who chose natural pregnancy was 16.67% (3/18), showing no statistical difference (*p* = 0.072).

**Conclusion:**

Molecular classification may be associated with hormone treatment response in patients with AEH, EC patients with POLEmut and p53wt had better progestin response, and those with MMRd and p53abn had the poorest response and the highest recurrence rate. Recurrence was common after CR, but close review is necessary. For further investigation of the role of molecular classification in fertility-sparing treatment of AEH/EC, larger prospective studies are necessary.

## 1 Introduction

Endometrial adenocarcinoma is the most commonly diagnosed gynecological cancer and ranks fourth in female malignancies in developed countries (1, 2). Atypical endometrial hyperplasia (AEH) is a precancerous stage of endometrial cancer (EC), with a 29% risk of progression to endometrial cancer (3). Unfortunately, in recent years, the incidence of young endometrial cancer has been rising; 4%–14% of women with endometrial cancer are younger than 40 years and want to preserve their fertility, and over 70% of them are nulliparous and have a strong wish to have children.

Although surgery is the standard treatment for endometrial cancer, which includes hysterectomy, bilateral salpingo-oophorectomy, and pelvic (or para-aortic) lymph node dissection, it also means a permanent loss of fertility while treating the disease. For patients who want to preserve fertility, fertility-preserving treatment is very important (4). The European Society of Gynaecological Oncology (ESGO), the European SocieTy for Radiotherapy & Oncology (ESTRO) and the European Society of Pathology (ESP) guidelines state that conservative treatment is considered for EC patients with stage IA, grade 1 and those without myometrial invasion or distant metastasis (5).

In recent decades, several studies have revealed that fertility-sparing treatment with progestin-based therapy provides promising results in patients with AEH and stage IA, grade 1 ECs. A study has shown that fertility-preserving treatment for AEH and stage IA, grade 1 EC patients was effective, achieving a complete disease remission rate of 84.5% and a pregnancy rate of 70.7% (6). Another study revealed that fertility-preserving treatment for young women with stage IA, grade 2 endometrial carcinoma was feasible, and 75% (3/4) of the patients had a complete response. Lago V’s study showed that the rate of complete response to fertility-sparing management was 74%, and 8.2% of patients presented a partial response. Additionally, 13 (17.8%) patients presented with persistent disease, and six (8.2%) relapsed after response. Levonorgestrel-releasing intrauterine system (LNG-IUS) was associated with a higher complete response rate than the other methods (87.2 vs. 58.8%; *p* = 0.01) (7). Unfortunately, a significant proportion of patients may experience disease progression, recurrence, or poor pregnancy outcomes (8).

In recent years, to assess the prognostic marker of the response to conservative treatment, several clinical, histological, and immunohistochemical markers such as PTEN, ARID1A, L1CAM, β-catenin, CTNNB1, and TP53 have been proven to be useful (9–11). Recent advances have shifted our understanding of endometrial cancer to molecular genetic features. Recently, the Proactive Molecular Risk Classifier for Endometrial Cancer (ProMisE) molecular classifier has shown prognostic value in endometrial carcinoma (12); for example, a study showed that TP53-mutated tumors were associated with poor prognosis, independently of the International Federation of Gynecology and Obstetrics (FIGO) stage and histological grade (13). Another study investigated the association of molecular subtype with progesterone response in patients with EC or AEH and included that molecular subtype may be associated with progesterone response in patients with EC/AEH. Copy number-low (CN-L) tumors had the best response, and microsatellite instability-high (MSI-H) tumors had the poorest (14). Unfortunately, the number of studies that have evaluated whether the ProMisE classification could provide important information on treatment choice for young women with low-grade, low-stage endometrial carcinoma wishing to preserve fertility is limited (15). Larger studies are needed to further investigate the role of molecular classification in the hormone management of AEH/EC.

On this account, the aim of this retrospective analysis was to further explore the predictive significance of molecular classification in the conservative treatment of EC and AEH.

## 2 Materials and methods

### 2.1 Study design and participants

Patients who were admitted to the First Affiliated Hospital of Zhengzhou University and the First Affiliated Hospital of Xinxiang Medical College between 1 January 2010 and 31 December 2022 were considered eligible if they met the inclusion criteria.

The inclusion criteria were as follows: 1) patients aged between 20 and 42 years who expressed a strong desire for fertility-sparing therapy; 2) patients diagnosed with histologically confirmed AEH or stage Ia, grade 1 adenocarcinoma, limited to the endometrium; 3) those who tested positive for progesterone receptor through immunohistochemical staining; 4) serum cancer antigen 125 (CA125) level was within the normal range; and 5) the medical records and pathology reports were consecutive.

Patients were excluded if they met any of the following criteria: 1) suspected myometrial invasion or extrauterine metastasis based on pelvic ultrasound or magnetic resonance imaging (MRI) in patients with EC; 2) presence of specific pathological types, such as low-differentiated adenocarcinoma, serous papillary carcinoma, or clear cell carcinoma; 3) the medical records were incomplete; 4) patients received hysterectomy; and 5) follow-up time <1 year.

### 2.2 Diagnosis and reassessment

Atypical endometrial hyperplasia or endometrial cancer was diagnosed by dilation and curettage or hysteroscopic endometrial biopsy. The paraffin-embedded slides were reevaluated in a blinded manner by two experienced gynecological pathologists (including at least one deputy chief pathologist) at the Obstetrics and Gynecology Hospital of Zhengzhou University to confirm the primary diagnosis. Immunohistochemistry was used to determine the expression of estrogen and progesterone receptors in endometrial specimens.

General patient information (including age, weight, height, treatment method, and history of pregnancy) and serum results (including cancer antigen 125 and human epididymis protein 4) were obtained from medical records. Body mass index (BMI) was calculated as weight (kg)/height (m^2^); overweight was defined as BMI ≥ 25 kg/m^2^, while obesity was defined as BMI ≥ 30 kg/m^2^.

### 2.3 Treatment and evaluation

Regarding the fertility-sparing treatment, the therapeutic regimens were considered one of the following: 1) at a dose of megestrol acetate (MA) 160–320 mg per day alone, 2) oral MA at a dose of 160–320 mg per day combined with metformin (850 mg, twice daily), or 3) oral MA at a dose of 160–320 mg daily combined with LNG-IUS intrauterine insertion.

Furthermore, the endometrial specimens were evaluated during fertility-sparing treatment every 3 months. The endometrial biopsy remission was recorded at 6, 9, and 12 months of fertility-sparing treatment. The primary endpoint of the study was the pathological complete response (CR) rate, defined as the proportion of patients with a CR among all patients. The secondary endpoints included remission rate, pregnancy rate, and pregnancy outcome. Treatment response was considered as follows: CR, absence of any cancerous or hyperplastic lesion; partial response (PR), presence of residual hyperplasia or carcinoma with incomplete degeneration or atrophy of endometrial glands; stable disease (SD), defined as the persistence of AEH or EC; and progressive disease (PD), which included progressions to a higher-grade lesion or clinically progressive disease, such as extrauterine disease or lymph node metastasis. Relapse is defined as a reappearance of EC or AEH after CR has been achieved during follow-up.

### 2.4 Molecular classification

The Cancer Genome Atlas (TCGA) performed an integrated genomic, transcriptomic, and proteomic characterization of 373 endometrial carcinomas using array- and sequencing-based technologies, and the results classified endometrial cancers into four categories: POLE ultramutated, microsatellite instability hypermutated, copy-number low, and copy-number high (16). Based on The Cancer Genome Atlas genomic subgroups, Talhouk A et al. (17) developed a molecular classification system, the ProMisE, which included polymerase-ϵ (POLE)-mutated (POLEmut), tumor protein 53 (p53) abnormal (p53abn), p53 wild type (p53wt), and mismatch repair deficiency (MMRd). In order to assess the association of endometrial categories and the response to fertility-sparing treatment, as well as pregnancy outcome, the histological sections of 118 patients were obtained from paraffin-embedded endometrial biopsy. First, sequencing was performed for POLE exonuclease domain mutations (POLE EDMs); if the POLE mutation and TP53 are present together, the tumor will be attributed to the POLE gene mutation. Second, to identify MMRd, immunohistochemistry (IHC) was performed for the presence or absence of mismatch repair (MMR) proteins MLH1, PMS2, MSH2, and MSH6. Third, p53 (p53wt and p53abn) was detected by IHC, or TP53 gene mutations were detected by next-generation sequencing (NGS). The patient was classified as having no specific molecular profile (NSMP) if the above three conditions were not found.

### 2.5 Follow-up

According to ESGO/ESHRE/ESGE guidelines (5), intensive follow-up to assess the endometrial response is needed; however, no clear and strict interval or assessment method for the follow-up of patients after fertility preservation in endometrial is available.

Most authors recommend endometrial biopsy every 3–6 months either by dilation and curettage or by hysteroscopic endometrial biopsy (18, 19). In the study, hysteroscopy and directed endometrial biopsy or dilation and curettage were performed every 3 months during treatment; pelvic ultrasound examination and contrast-enhanced MRI were also suggested. The success of the fertility-sparing treatment was considered when there were two consecutive complete response endometrial biopsies with a minimal interval of 3 months, and pregnancy was recommended or maintenance treatment was offered.

For patients who achieved complete response, strict surveillance, which includes endometrial sampling biopsies and transvaginal ultrasound or MRI, was required every 3 to 6 months to detect relapse. Assisted reproductive technology was recommended to achieve pregnancy in women who achieved CR.

### 2.6 Statistical analysis

Once the data were collected, statistical analyses were conducted using IBM SPSS Statistics for Windows (Version 26.0; IBM Corp., 2019). Categorical data were compared using the chi-square and Fisher’s exact tests, whereas means were calculated for continuous variables and compared between groups using the t-test. Statistical significance was set at *p* < 0.05.

## 3 Results

### 3.1 Patient characteristics

A total of 118 EC or AEH patients with complete clinical, pathological, and outcome data were enrolled in this study; 2 patients whose endometrial samples were not enough to molecular classify were excluded, and 116 patients were included in this study ultimately. The baseline characteristics are summarized in **Table 1**. They were then divided into four subgroups according to the molecular analyses by immunohistochemistry and single gene sequencing. A total of 10 (8.60%) patients had POLE gene mutations. There were 81 (69.83%) patients in the p53wt/NSMP subgroup, accounting for the largest proportion, 9 (7.75%) patients in the p53abn subgroup, and 16 (13.79%) patients in the MMRd subgroup.

**Table 1:** Primary clinicopathologic characteristics of all patients.

Variable	POLEmut (n = 10)	NSMP/p53wt (n = 81)	P53abn (n = 9)	MMRd (n = 16)	Total (n = 116)	*P*-value
Age (years)						
Mean ± SD	35.10 ± 2.42	33.16 ± 3.27	34.22 ± 3.49	32.31 ± 4.22	33.29 ± 3.40	0.176
BMI (kg/m^2^)						
Mean ± SD	25.40 ± 2.02	26.80 ± 2.81	25.37 ± 2.77	27.57 ± 2.77	26.68 ± 2.77	0.115
Diagnostic procedure						
D&C	3 (30.0%)	15 (18.5%)	2 (22.2%)	4 (25.0%)	24 (20.7%)	-
HSC + D&C	7 (70.0%)	66 (81.5%)	7 (77.8%)	12 (75.0%)	92 (79.3%)	
No. of pregnancies						0.783
0	6 (60.00%)	60 (74.07%)	6 (66.67%)	11 (68.75%)	83 (71.55%)	
≥1	4 (40.00%)	21 (25.93%)	3 (33.33%)	5 (31.25%)	33 (28.45%)	
Treatment method						0.703
MA	6 (60.00%)	42 (51.85%)	3 (33.33%)	8 (50.00%)	59 (50.86%)	
MA + MET	3 (30.00%)	18 (22.22%)	4 (44.44%)	5 (31.25%)	30 (25.86%)	
Lng IUD + MA	1 (10.00%)	21 (25.93%)	2 (22.22%)	3 (18.75%)	27 (23.28%)	
ER expression: N (%)						0.636
Negative	0 (0%)	2 (2.47%)	0 (0%)	0 (0%)	2 (1.73%)	
Positive	10 (100%)	79 (97.53%)	9 (100%)	16 (100%)	114 (98.27%)	
PgR expression: N (%)						0.577
Negative	1 (10.00%)	3 (3.70%)	0 (0%)	0 (0%)	4 (3.45%)	
Positive	9 (90.00%)	78 (96.29%)	9 (100%)	16 (100%)	112 (96.55%)	
PCOS: N (%)	0 (0%)	16 (19.75%)	0 (0%)	1 (6.25%)	17 (14.65%)	0.034
Diabetes	2 (20.00%)	12 (14.81%)	2 (22.22%)	3 (18.75%)	19 (16.38%)	0.445
E2 (pg/mL)	157.5 (20.0–604.0)	159.0 (18–617.0)	135.0 (20.0–6060)	161.5 (32.0–565.0)	160.0 (18.0–7617.0)	0.903
P (ng/mL)	0.78 (0.26–21.77)	1.42 (0.21–38.99)	1.19 (0.56–20.94)	1.37 (0.43–20.21)	1.32 (0.20–38.99)	0.799
T (ng/dL)	13.10 (5.04–47.31)	28.40 (5.79–64.96)	11.68 (8.21–41.12)	16.24 (7.79–50.46)	26.53 (5.04–64.97)	0.006
Day 2–5 FSH (mIU/mL)	5.09 (4.32–8.42)	5.53 (1.48–8.66)	3.45 (2.46–8.71)	4.98 (2.72–8.58)	5.31 (1.48–8.71)	0.500
Hyperlipidemia: N (%)	5 (50.0%)	58 (71.6%)	6 (66.7%)	11 (68.8%)	80 (69.0%)	0.605
CA125	16.87 ± 8.72	18.44 ± 5.45	23.02 ± 10.82	18.99 ± 8.08	18.73 ± 6.63	0.193

POLEmut, polymerase-ϵ-mutated; NSMP, no specific molecular profile; p53wt, tumor protein 53 wild type; p53abn, p53 abnormal; MMRd, mismatch repair deficient; BMI, body mass index; D&C, dilation and curettage; HSC, hysteroscopy; MA, megestrol acetate; PCOS, polycystic ovary syndrome; CA125, cancer antigen 125. MET, Metformin; Lng IUD, Levonorgestrel-releasing Intrauterine Device; ER, Estrogen Receptor; PgR, Progesterone Receptor; FSH, the Follicle-stimulating hormone.

In our study cohort, the median age was 33 (range 22.0–42.0) years, the median BMI was 26.515 kg/m^2^ (range 19.05– 37.10 kg/m^2^), and approximately 83 (71.55%) patients had no history of pregnancy at the time of treatment. Approximately 98.27% of patients had positive estrogen receptor expression, and 96.55% of patients had progesterone receptor expression before the primary administration of hormone therapy. The mean CA125 level of our study cohort was 18.73 ± 6.63, and there was no difference among the four molecular types (*p* > 0.05). Nineteen patients were complicated with diabetes and 17 (14.65%) cases with polycystic ovary syndrome (PCOS). There was no significant difference in estrogen/progesterone expression and lipid levels prior to the first administration of hormone therapy among the four molecular types (*p* > 0.05). However, there were statistical differences in testosterone levels, and p53wt has the highest testosterone level (*p* < 0.05).

Overall, 59 (50.86%) patients in our study cohort received MA as the fertility-sparing treatment, 30 (25.86%) received a combination therapy of MA and metformin, and another 27 (23.28%) patients received a combination therapy of MA and Levonorgestrel-releasing Intrauterine Device (Lng IUD). The mean treatment duration of our cohort was 10.39 ± 2.33, and the median follow-up time was 36.9 months, ranging from 12.8 to 86.8 months.

### 3.2 Association of response with molecular classification

**Figure 1:**
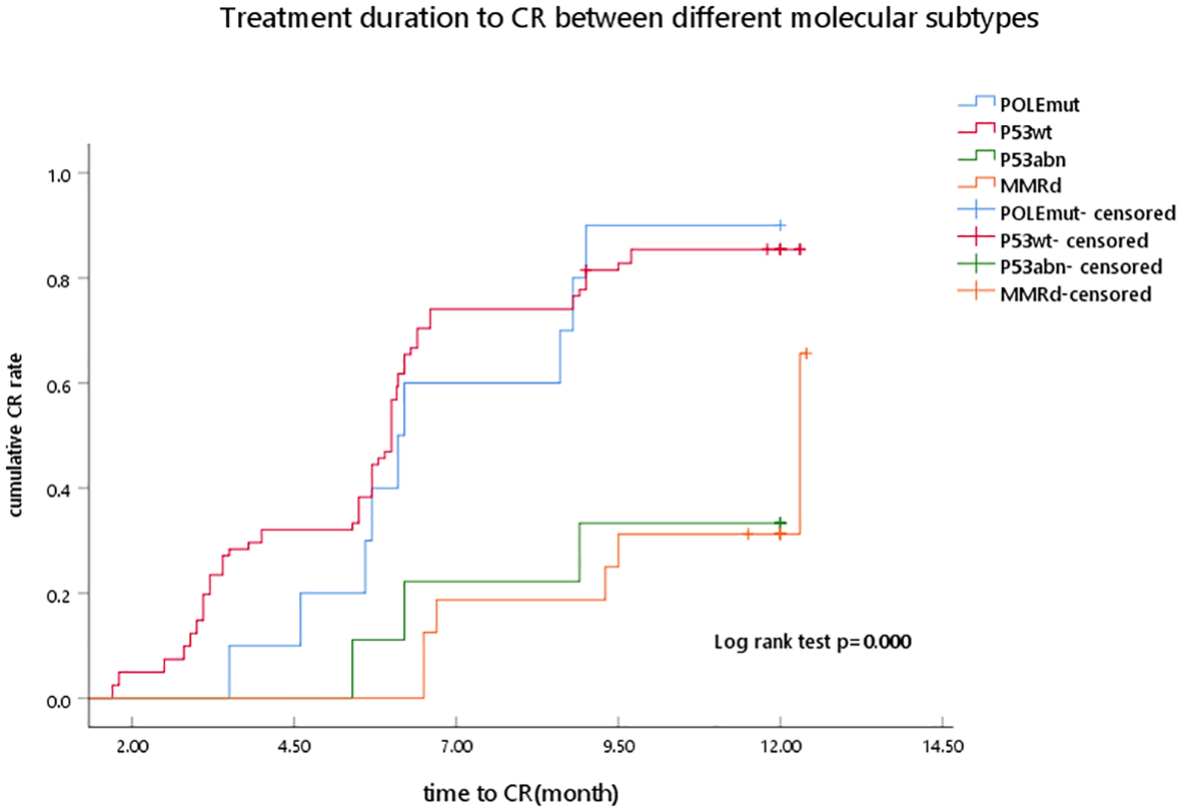
Treatment duration to CR between different molecular subtypes as determined by Kaplan–Meier analysis and compared by the log-rank test. POLEmut, DNA polymerase epsilon mutation; p53wt, p53 wild type; p53abn, p53 abnormal; MMRd, MMR deficiency; CR, complete response.

**Table 2** and **Figure 1** show the associations between molecular classification and fertility-sparing treatment outcomes. A total of 71 AEH and 45 EC cases completed the fertility-sparing treatment, and the overall CR rate was 61.21%, 73.27%, and 75.00% at 6, 9, and 12 months of fertility-sparing treatment, respectively. At 6, 9, and 12 months of fertility-sparing treatment, the cumulative CR rate was 70.00%, 90.00%, and 90.00% in the POLEmut subgroup, respectively, and 72.84%, 85.19%, and 85.19% in the p53wt subgroup respectively, while the cumulative CR rate was 22.22%, 22.22%, and 33.33%, respectively, in the p53abn subgroup. In the MMRd subgroup, the cumulative CR rate was 31.25%, 31.25%, and 37.50%, respectively, at 6, 9, and 12 months of fertility-sparing treatment. The MMR-deficient and p53abn cases showed lower CR rates than the POLEmut and p53wt cases, with significant statistical differences (*p* < 0.01). Of the 71 AEH patients, 56 (78.87%) cases achieved CR, while the CR rate was 68.89% (31/45) in EC cases, and there was no correlation between CR rate and pathological type (*p* = 0.616).

**Table 2:** Response distribution in the four subgroups in the study.

Variables	All patients	POLEmut	NSMP/P53wt	P53abn	MMRd	t/F/X^2^	*P*-Value
Pathology	116	10	81	9	16		
AEH	71	6	51	5	10		
EC	45	4	30	4	6		
6-month CR rate							
All	71 (61.21%)	7 (70.00%)	59 (72.84%)	2 (22.22%)	3 (18.8%)	15.791	<0.001
AEH	45 (63.38%)	4 (66.67%)	38 (74.51%)	1 (20.00%)	2 (20.00%)	11.212	0.001
EC	26 (57.78%)	3 (75.00%)	21 (70.00%)	1 (25.00%)	1 (16.66%)	6.343	0.012
9-month CR rate							
All	85 (73.27%)	9 (90.00%)	69 (85.19%)	2 (22.22%)	5 (31.25%)	16.874	<0.001
AEH	54 (76.06%)	6 (100.00%)	44 (86.27%)	1 (20.00%)	3 (30.00%)	15.563	<0.001
EC	31 (68.89%)	3 (75.00%)	25 (83.33%)	1 (25.00%)	2 (33.33%)	2.939	0.086
12-month CR rate							
All	87 (75.00%)	9 (90.00%)	69 (85.19%)	3 (33.33%)	6 (37.50%)	15.680	<0.001
AEH	56 (78.87%)	6 (100.00%)	44 (86.27%)	2 (40.00%)	4 (40.00%)	10.159	0.001
EC	31 (68.89%)	3 (75.00%)	25 (83.33%)	1 (25.00%)	2 (33.33%)	4.656	0.031
12-month PD rate							
All	19 (16.40%)	1 (10.00%)	8 (9.90%)	4 (44.40%)	6 (37.50%)	11.191	0.011
AEH	8 (11.1%)	0 (0%)	4 (7.8%)	1 (20%)	3 (50.00%)	5.189	0.023
EC	11 (25.00%)	1 (25.00%)	4 (13.30%)	3 (75.00%)	3 (50.00%)	8.610	0.035
2-year recurrence rate							
All	23 (26.40%)	3 (33.33%)	12 (17.39%)	3 (100.00%)	5 (83.33%)	10.111	0.001
AEH	14 (25.00%)	2 (33.33%)	7 (15.91%)	2 (100.00%)	3 (75.00%)	12.287	0.006
EC	9 (29.03%)	1 (33.33%)	5 (20.00%)	1 (100.00%)	2 (100.00%)	7.763	0.051
Median treatment duration to CR (months)							
All	5.95	6.1	5.7	6.3	9.4	6.576	<0.001
AEH	5.90	6.2	5.7	8.9	7.95	4.421	0.008
EC	6.00	6.5	5.7	–	9.7	5.799	0.024
Median duration to recurrence (months)							
All	15.8 (5.9–79.7)	17.9 (15.8–20.6)	23.7 (7.8–79.7)	8.9 (7.9–13.7)	9.8 (5.9–13.7)	2.117	0.132
AEH	17.65 (7.8–9.7)	18.2 (15.8–0.6)	25.7 (7.8–79.8)	11.3 (8.9–3.7)	9.8 (8.6–13.7)	0.719	0.563
EC	13.6 (5.9–35.8)	–	17.8 (9.7–35.8)	–	9.75 (5.9–13.6)	0.779	0.554
Treatment duration (months)	10.39 ± 2.33	10.81 ± 3.36	12.11 ± 6.34	12.71 ± 4.21	11.14 ± 3.74	2.523	0.061
Follow-up duration (months)	36.9 (12.8–86.8)	47.1 (12–108)	23.7 (14.6–75.4)	45.65 (19.7–88.5)	43.95 (12–108)	2.585	0.057

POLEmut, polymerase-ϵ-mutated; NSMP, no specific molecular profile; p53wt, tumor protein 53 wild type; p53abn, p53 abnormal; MMRd, mismatch repair deficient; AEH, atypical endometrial hyperplasia; EC, endometrial cancer; CR, complete response.

### 3.3 Progression and molecular classification

At the 12-month evaluation, one patient’s (10.00%) endometrial pathology showed PD and was withdrawn from the study in the POLEmut subgroup, she received a laparoscopic total hysterectomy plus bilateral salpingo-oophorectomy plus pelvic lymphadenectomy, and grade 2 with myometrial invasion was detected in the final pathological examination.

In patients with P53wt, nine cases [11.11%, SD (n = 1) and PD (n = 8)] showed treatment failure in the initial response to progestin treatment, and eight patients underwent immediate hysterectomy plus bilateral salpingo-oophorectomy and pelvic lymph node dissection because of treatment failure. Of the patients, eight (9.87%) showed PD and received surgery, one patient presented superficial myometrial invasion, and no tumor was found in the final uterus specimen. The remaining surgical specimens were compatible with atypical endometrial hyperplasia and stage I, grade 1 endometrial carcinoma.

For patients with p53abn, four (44.44%) patients showed PD, and the final surgical treatment was performed. Extra-uterine metastasis was detected for one surgical specimen. Lymphovascular space invasion was detected for one patient, and two surgical specimens presented myometrial invasion >50%.

For patients with MMRd, seven cases [SD (n = 1) and PD (n = 6)] showed no response to progestin treatment, and six patients underwent immediate surgical treatment, which included hysterectomy plus bilateral salpingo-oophorectomy and bilateral pelvic lymph node dissection because of treatment failure. Histopathological analysis revealed deep myometrial invasion (>50%), with concurrent ovarian metastasis documented in one patient, and the remaining two patients presented stage I, grade 2 endometrial carcinoma.

**Figure 2:**
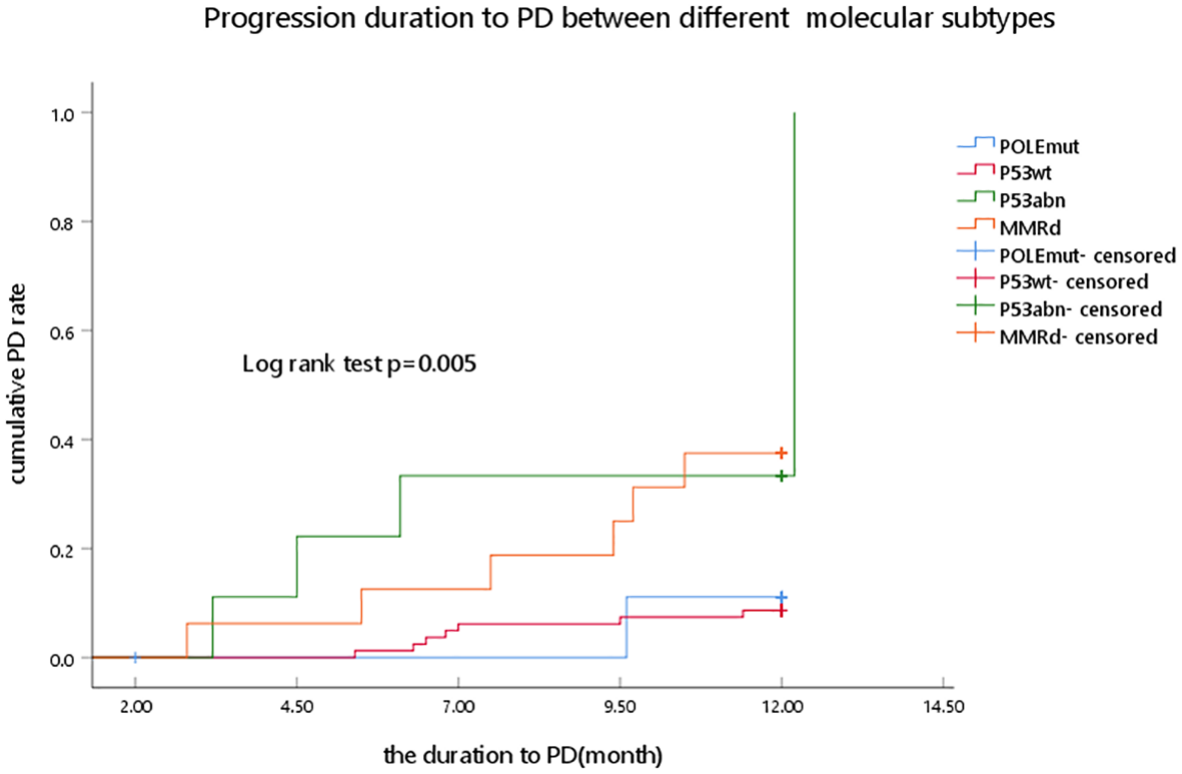
Progression duration to PD between different molecular subtypes as determined by Kaplan–Meier analysis and compared by the log-rank test. POLEmut, DNA polymerase epsilon mutation; p53wt, p53 wild type; p53abn, p53 abnormal; MMRd, MMR deficiency; PD, progression disease.

In the presented study, the p53abn and MMRd subgroups had a higher PD rate than the POLEmut and P53wt subgroups (44.40% and 37.50% vs. 10.00% and 9.90%, respectively), and a significant statistical difference was observed (*p* = 0.011) (**Figure 2**).

### 3.4 The treatment response time

The duration from the start of treatment to achieve CR was defined as CR time, the median treatment duration to CR was 5.95 months (1.6–12.9 months), and there was a statistically significant difference between the four groups (*p* < 0.001). For AEH patients, the median CR duration was 8.9 and 7.95 months in the p53abn and MMRd subgroups, respectively, which had a higher CR duration than the POLEmut and p53wt subgroups (6.2 and 5.7 months), and a statistical significance was observed (*p* < 0.001). For EC patients, the MMRd subgroup had a higher median CR time than the POLEmut and p53wt subgroups (9.7 vs. 6.2, 5.7 months), and there were statistically significant differences (*p* = 0.024) (**Table 2**).

### 3.5 Follow-up and relapse

**Figure 3:**
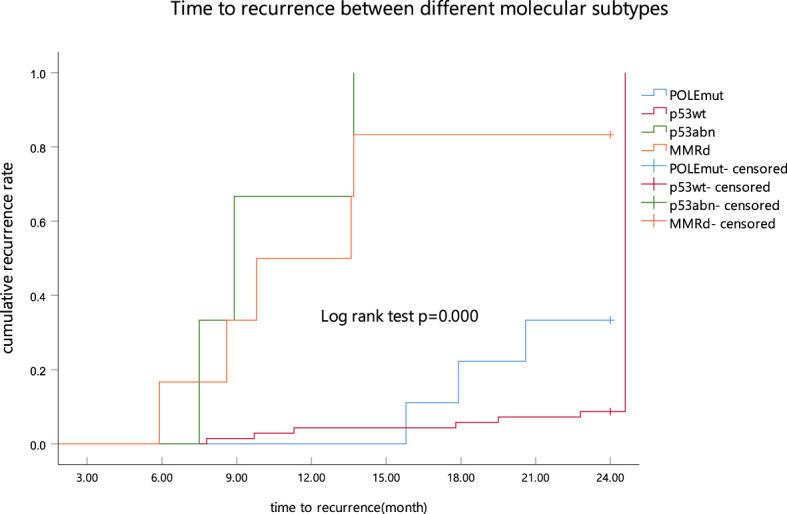
Time to recurrence between different molecular subtypes as determined by Kaplan–Meier analysis and compared by the log-rank test. POLEmut, DNA polymerase epsilon mutation; p53wt, p53 wild type; p53abn, p53 abnormal; MMRd, MMR deficiency.

The mean follow-up time in the present study was 31.74 ± 11.51 months (range, 12.8–86.8 months). At 24 months of follow-up, 23 (26.44%) patients experienced disease recurrence among 87 patients who achieved CR. A statistical significance was found among the four groups (*p* < 0.01), with the p53abn subgroup having the highest cumulative recurrence rate after CR (3/3) (**Figure 3**). The median duration from CR to recurrence was 11.3 months (range 8.9–13.7 months) (**Table 2**).

### 3.6 Outcome of pregnancy

Among the 87 patients who achieved CR in four subgroups, 56 (64.37%) patients attempted pregnancy, and the pregnancy rate of 38 patients who received *in vitro* fertilization-embryo transfer was 47.37% (18/38), resulting in eight live births; the pregnancy rate of 18 patients who chose natural pregnancy was 16.67% (3/18), resulting in one live birth. There was no statistical difference between the two methods of conception (*p* = 0.072).

## 4 Discussion

EC was among the most frequently occurring malignancies in developed countries, ranking fourth among female malignant tumors. According to the literature, the incidence of EC was 23.5 cases per 100,000 women (20). Of these patients, 25% were premenopausal, with 2.5% to 14.4% diagnosed before the age of 40 years (21). Due to abnormal uterine bleeding as a common symptom, EC was usually diagnosed early, with diagnostic curettage easily providing tissue samples. Moreover, 84% of type I endometrioid adenocarcinoma cases were well-differentiated (22). For young women with high-differentiation early-stage, non-metastatic EC or AEH, fertility-sparing treatment was an option for the patients who were selected based on a thorough evaluation of reproductive potential (5, 23).

Over the past few years, significant progress has been made in the fertility-preserving treatment of endometrial cancer and AEH, and many biomarkers for screening suitable fertility-preserving treatment candidates have been studied to determine their relevance to the risk and outcome of early endometrial cancer (24, 25).

In recent years, molecular classification has become more and more valuable in guiding the treatment and prognosis evaluation of endometrial cancer, but there has been no consensus. Xiaofeng Lv et al. (26) reported a study of 93 AEH or early-stage EC patients who received LNG-IUS to preserve fertility. In their cohort, among the 93 patients, 15 (16.1%) were classified as MMRd, 6 (6.5%) as POLE mutated, 5 (5.4%) as p53 abnormal, and 67 (72.0%) as p53wt. Patients with the p53 abnormal subtype had the lowest overall CR rate (40%) and the highest recurrence rate (2/2). Another study demonstrated that patients with POLE mutations had the highest disease progression rate (50.0%, *p* = 0.013), while the MSI-H group had the highest recurrence rate (50.0%, *p* = 0.042) (27).

Christian Dagher et al. (14) reported the outcomes of 20 EC and AEH patients in their institution with 16 (80%) CN-L tumors, 3 (15%) MSI-H tumors, and 1 (5%) POLE-ultramutated tumor; CN-L tumors had a 62% of CR rate and 19% of PD rate; for MSI-H tumors, 33% of patients had SD and 66% of patients had PD; for POLE ultramutated tumors, one patient had PD. The study indicated that the molecular subtype may be associated with progesterone response in patients with EC/AEH. CN-L tumors had the best response, and MSI-H tumors had the poorest. Puechl AM et al. (28) classified 58 endometrial cancer or endometrial intraepithelial neoplasia (EIN) into four groups as per the ProMisE: 44 patients (75.9%) were classified as p53wt, 6 (10.3%) as MMRd, 4 (6.9%) as p53abn, and 4 (6.9%) as *POLE*-mutated. Of the 58 patients, the median time to progression or definitive therapy was 7.5 months, with p53abn tumors having the shortest time to progression or definitive therapy.

In our study, the ProMisE molecular classifications were performed for all evaluated endometrial specimens. Of 116 patients, 10 (8.62%) were classified as POLEmut, 81 (69.82%) as p53wt, 9 (7.76%) as p53 abnormal, and 16 (13.76%) as MMRd, which was in agreement with the literature report (14, 28). In the present study, patients with the p53 abnormal and MMRd subtypes had a lower CR rate and a higher progression rate than the POLEmut and p53wt subgroups at 6, 9, and 12 months of evaluation. In a study by Antonio Raffone et al., immunohistochemistry for MMR was performed on 69 women, deficient MMR expression was observed in 8.7% of cases, the author reported that resistance to conservative treatment and recurrence were more common in MMR-deficient than MMR-proficient cases, and a deficient immunohistochemical expression of MMR could predict recurrence after fertility-sparing treatment (22, 24–29). Young Shin Chung et al. (8) demonstrated that patients with mismatch repair deficiency had a significantly lower complete response or partial response rate than those with wild-type p53 in terms of the best overall response (44.4% vs. 82.2%) and complete response rate at 6 months (11.1% vs. 53.3%; four of nine patients underwent immediate hysterectomy, and three presented upstaged diagnosis after hysterectomy. In another study by Puechl AM et al. (28), in patients with MMRd or *POLE*-mutated tumors, 33.3% and 25% progressed or required definitive therapy, respectively, regardless of histology; patients with p53abn tumors had the shortest time to progression or definitive therapy. Future prospective studies in patients with MMRd tumors should further elucidate the prognostic value of MMRd in conservative therapy. M Zakhour et al. (30) reported that patients with MMRd had a higher incidence of invasive cancer and a lower incidence of resolution with progestin therapy.

In the present study, p53abn had the highest incidence of progression (4/9, 44.40%) and recurrence rate (3/3, 100%). In a retrospective study by Hongfa Peng et al. (31), they evaluated 51 patients with AEH who underwent fertility-sparing treatment, patients with p53abn had higher relapse rates than those with p53wt at the 1- and 2-year follow-ups after achieving CR; moreover, patients with p53abn had a higher incidence of disease progression at 3 and 4 years after fertility-sparing treatment. Xu Y et al. (27) retrospectively investigated 90 patients who received fertility-sparing treatment, patients with POLE mutations had the highest disease progression rate (50.0%, *p* = 0.013), and the MSI-H group had the highest recurrence rate (50.0%, *p* = 0.042).

In our study, the p53abn subgroup had the worst fertility-sparing effect among the four subgroups, followed by the MMRd subgroup. The p53abn subgroup had the longest duration to CR and the shortest duration to recurrence, followed by the MMRd subgroup. However, the present study also had certain limitations that need to be considered. First, given the limitation of retrospective analysis and the small number of patients in the p53abn and MMRd subgroups, further prospective studies with larger sample sizes are needed to further strengthen our conclusions. However, our study should not be neglected for the detailed data recording and strict adherence to inclusion and exclusion criteria for every AEH or EC patient, which avoided selection bias.

There were many different types of medical treatment that could be performed for women with AEH or stage IA, grade 1 EC. While continuous progestin-based therapy has been traditionally the cornerstone of fertility-sparing treatment, a meta-analysis evaluated the safety and efficacy of the available medical treatment and concluded that fertility-sparing treatment was a safe method of management in young women with endometrial cancer/atypical endometrial hyperplasia (32). Medroxyprogesterone acetate and megestrol acetate are the most used progestins (33). Medroxyprogesterone acetate (MPA; 400–600 mg/day) or MA (160–320 mg/day) could be administered orally every day (34). However, in a meta-analysis by Lucchini SM (35), megestrol acetate was shown to result in higher remission probabilities compared to MPA and other hormone treatments, which may be due to its higher bioavailability of MA compared to MPA following oral administration. Another alternative way of progestin administration was the use of a levonorgestrel intrauterine device, which, combined with oral progestins, has been emerged as the frontrunner in improving the complete response (CR) (SUCRA=98.7%), objective response rate (ORR) (SUCRA=99.1%), pregnancy rate (SUCRA=83.7%), and mitigating progression (SUCRA=8.0%) and relapse rate (SUCRA=47.4%) and decreasing the likelihood of adverse events (SUCRA=4.2%) (36). Some studies evaluated the efficacy of metformin in MA-based fertility-sparing treatment and concluded that metformin plus MA was associated with a higher early CR rate compared with MA alone in AEH patients. Moreover, metformin may be more efficacious for patients with BMI ≥ 25 kg/m^2^ (37, 38). In the present study, 59 (50.86%) patients were treated with MA alone, 30 (25.86%) cases received MA combined with LNG-IUS, and 27 (23.28%) cases were treated with MA combined with metformin, although the higher CR rate was observed in the combination group, but there was no statistical difference among three methods.

A growing number of studies in addition to the above studies have focused on the relationship between molecular classification and treatment prognosis of endometrial carcinoma, whether in fertility-preserving treatment for early-stage EC or surgical treatment for advanced EC. Unfortunately, there are no randomized controlled trials comparing the different types of medical treatment in women. According to the literature, in the younger age group with low-grade, stage IA endometrial carcinomas, the greatest benefit of progestin management was seen in women harboring p53 wild-type tumors. For example, Britton H et al. (39) assessed the prognostic significance of the ProMisE in young (<50 years) women with EC and demonstrated that the ProMisE maintained a strong association with overall, disease-specific, and progression-free survival on multivariable analysis. Other studies have shown that TP53-mutated tumors were associated with poor prognosis, independently of the FIGO stage and histological grade and independently of clinical risk of relapse (40). Among patients with FIGO stage I–II tumors, six (38%) TP53-mutated tumors had a low/intermediate clinical risk of relapse (13, 40). Peng S et al. (41) investigated the prognostic significance of molecular classification on treatment outcomes of fertility-sparing treatment (FST) in early-stage EC and concluded that POLE EDM patients tended to obtain promising outcomes. MMR-D cases should be cautiously administrated for FST with close surveillance. Patients with p53wt demonstrated favorable outcomes, including those with superficial MI or G2 EC.

In our retrospective study, patients in the p53abn and MMRd subgroups had poorer responses to progesterone therapy, and relapse was more common than in the other subgroups; it should be further confirmed whether fertility-sparing treatment is suitable. Owing to the limited number of studies, for POLE-mutated carcinomas, the treatment choice in the conservative era is still unclear (5), while in our study, the POLE-mutated and P53wt subgroups had a good prognosis. In addition, although the pregnancy rate between *in vitro* fertilization-embryo transfer and natural pregnancy had no statistical difference, assisted reproduction should be performed as soon as possible after CR.

## Data Availability

The original contributions presented in the study are included in the article/supplementary material. Further inquiries can be directed to the corresponding author.
